# Human, Animal and Planet Health for Complete Sustainability

**DOI:** 10.3390/ani11051301

**Published:** 2021-04-30

**Authors:** Giuseppe Bertoni

**Affiliations:** Department of Animal Sciences, Food and Nutrition, Faculty of Agriculture, Food and Environmental Science, Università Cattolica del Sacro Cuore, 29122 Piacenza, Italy; giuseppe.bertoni@unicatt.it

**Keywords:** sustainability, animal health, human health, environment

## Abstract

**Simple Summary:**

Animal breeding must be seen within sustainability that includes human health and the health of the planet (ecology), without neglecting the economic and ethical aspects. The relationship with human health is dual, since in the absence of food of animal origin there is malnutrition, but excesses increase non-communicable diseases. However, animal farming is considered a cause of serious environmental impact, as well as a cause of suffering for animals (ethics). Therefore, it is proposed to modify the diet in relation to foods of animal origin, properly reducing them in rich countries, but increasing them in poor ones. To reduce the environmental impact of the animals, they must be reduced in number, but the quantities of food needed imply an increase in productivity and efficiency. Their good health is fundamental for these last objectives: to fight infectious and parasitic diseases, but also to ensure optimal feeding and living conditions to guarantee their welfare.

**Abstract:**

In order to discuss the concepts of animal health and sustainability, we must remind ourselves that ASF (animal source foods) can play a large role in human health, but that animals are assumed to have a negative role in the environment. Indeed, ASF can compromise human health, both in excess and in deficiency, so a proper amount of them is important. In addition, the environmental impact of farmed animals: land occupation, greenhouse gas (GHG) emissions, energy use and water utilization, acidification and eutrophication, must be minimized by reducing ASF consumption, as well as by increasing productivity. To achieve this, besides genetics, feeding and good management, the hygienic-sanitary and comfort conditions that ensure good health and welfare are essential. Impaired animal health can cause zoonosis and food-borne diseases and be responsible for economic and socio-economic losses (lower production-productivity and profitability) with consequential effects on the planet’s health too, and there are big differences between developing and developed countries. In the former, a prevalence of endemic infectious diseases and parasites is observed, and there is a lack of tools to restrain them; in the latter there is a decline of the above diseases, but an increase of stress-related diseases. Their reduction is equally important but requires a different strategy. In developing countries, the strategy should be to facilitate the availability of prevention and treatment means, while in developed countries it is necessary to use drugs correctly (to reduce residues, especially antimicrobials which are associated with important resistance risks to antibiotics) and improve the living conditions of animals (welfare).

## 1. Introduction

Such a title in not easily understandable if so called “ONE HEALTH” is not considered; namely, the innovative strategy to promote multi-sectoral and interdisciplinary application of knowledge and skills of medical, public health, veterinary and environmental experts by working together to address animal, human and environmental health challenges, to help in the search for solutions to the problems of mankind. In short, a strategy to attain optimal health for people, animals and the environment. This premise is important because, besides animal health per se, it includes the relationship between animal source foods (ASF) and human health, as well as the risks for the environment associated with animal breeding.

In fact, although sustainability is often considered to be “*the needs of the present without compromising the ability of future generations to meet their own needs*” [1], with particular attention to the environment, this word actually has a wider meaning. According to Drewnowsky [2] *the FAO (Food and Agriculture Organization) definition of sustainable diets has four dimensions: (1) nutrition and health, (2) economic, (3) social and cultural, and (4) environmental*. The new dimension “nutrition and health” is well explained by Burlingame and Dernini [3]: “*Sustainable diets are those diets with low environmental impacts that contribute to food and nutrition security and to healthy life for present and future generations. Sustainable diets are protective and respectful of biodiversity and ecosystems, culturally acceptable, accessible, economically fair and affordable, nutritionally adequate, safe, and healthy, while optimizing natural and human resources*”. None of the above “pillars” must be overlooked and a compromise is needed for a balanced overall approach. Despite this, we shall temporarily limit ourselves to the two pillars that mostly concern our topic: the role of ASF in the human diet and the environmental impact of their production (or agriculture as a whole).

The aim is, therefore, to better understand the true role of ASF for human health and to garner whether farmed animals are really responsible for natural system degradation. In other words, there are two questions: (i) can we reduce (or block) human ASF consumption without triggering health problems? (ii) is the consequent reduction (or disappearance) of bred animals sufficient to reverse the environmental impact of agriculture?

## 2. Role of Animal Source Foods (ASF) in Human Health

In recent years, numerous studies have claimed that a diet without ASF is preferable and that a healthy diet: “…largely consists of vegetables, fruits, whole grains, legumes, nuts, and unsaturated oils, includes a low to moderate amount of seafood and poultry, and includes no or a low quantity of red meat, processed meat, added sugar, refined grains, and starchy vegetables.” [4]. Such a diet is believed to avoid some of the negative effects related to diets in developed countries: obesity and non-communicable diseases, including coronary heart disease, stroke, and diabetes. Nevertheless, with the same purpose, other authors [5] simply suggest: “*moderation in unprocessed red meats, poultry, eggs, and milk and fewer processed (e.g., sodium-preserved) meats*” to prevent cardiovascular diseases (CVD) obesity and type 2 diabetes. Furthermore, the 3rd Report of the World Cancer Research Fund [6] has suggested: “*if you eat red meat, limit consumption to no more than about three portions per week. Three portions are equivalent to about 350 to 500 g cooked weight of red meat. Consume very little, if any, processed meat. The guidance on the maximum amount of red meat to eat is for the weight of the meat as eaten; a rough conversion, 500 g of cooked red meat is about equivalent to 700 to 750 g of raw meat* (carcasses as in OECD (Organisation for Economic Co-Operation and Development) [7]. *A diet low in red and processed meat does not have to exclude eggs, dairy, poultry or fish, and it allows for higher consumption of wholegrains, non-starchy vegetables, fruit and pulses (legumes) such as beans and lentils*.” The importance of these statements is magnified if the suggestions of the former WCRF report [8] are also considered; the limits placed on the consumption of red meat—in order not to run the risk of cancer—were 70 g/day, compared to the current 85. Going back to the 2018 report [6], it suggests that eating patterns including a lower intake of meat, processed meat and processed poultry are associated with reduced risk of CVD and type 2 diabetes in adults.

Therefore, it seems possible to assume that some authors take into account not the negative effects of the ASF, but only the negative effects of their excessive consumption. Salter [9] appears much more honest suggesting that the excessive levels of ASF consumption seen in many of the world’s wealthiest countries impacts on susceptibility to a range of chronic diseases, including obesity, diabetes, CVD and some cancers, as well as placing an unsustainable burden on the environment. Another evaluation, by Clark et al. [10], suggests that adult health is negatively impacted by the consumption of an additional serving of food per day (1 serving more than the cohort average) for 15 food groups. This seems arguable because it can be presumed that the selected cohorts are from developed countries and it is therefore likely that an additional serving of ASF above the norm will often represent an additional excess.

At the same time, Willet et al. [4], who only consider a vegetarian diet to be healthy, affirm in Panel 3: “*Because many regions, such as sub-Saharan Africa, still face severe burdens of undernutrition and malnutrition, and growing children often do not obtain adequate quantities of nutrients from plant source foods alone, source foods should be examined carefully*.” This perfectly corresponds to personal experience gained in the tribal area of India and in the Democratic Republic of Congo, supporting the idea that a much more balanced approach is needed, as recently suggested by Mozaffarian [11]: “*The ‘double burden’ of undernutrition and chronic diseases causes enormous economic losses and lost human potential across the lifespan. Globally, poor nutrition is responsible for 41% of all deaths (3.2 million per year from child and maternal undernutrition, 10.9 million per year from chronic diseases) and 48% of lost quality-adjusted life years (327 and 255 billion per year, respectively)*.” The poor nutrition that concerns about 3 billion people is very often related to few ASF: Randolph et al. [12] highlights that “*…livestock can also be used to deliver critical micronutrients needed to enhance the nutritional status of household members and secure their most fundamental livelihood asset, their human capital, as a pre-condition for alleviating poverty*.” Apparently, not everyone knows that malnutrition in children, especially in the first critical 1000 days from conception, also impairs their cognitive development [13], whereas providing essential nutrients through ASF can help ensure normal physical and cognitive development [14]. Indeed, Adesogan et al. [15] have recently quoted the World Health Organization for having described animal-source foods as “*the best source of high-quality nutrient-rich food for children aged 6–23 months*”. The same authors added that “*Livestock and ASF are vital to sustainability as they play a critical role in improving nutrition, reducing poverty, improving gender equity, improving livelihoods, increasing food security, and improving health*.” However, this said, vegetarianism or veganism may well be nutritionally feasible in the very places where ASF are overconsumed, and a large variety of plant foods and micronutrient supplements are typically available. The opposite is true in poor countries where, according to FAO [16]: “*Even small amounts of animal source foods can improve the nutritional status of low-income households. Meat, milk and eggs provide proteins with a wide range of amino acids as well as micronutrients such as iron, zinc, vitamin A, vitamin B12, and calcium, in which many malnourished people are deficient*”.

Therefore, it can be concluded that, although it may be corrected to avoid ASF excesses in rich countries in order to avoid the risk of developing chronic diseases such as cancer and diabetes, it is instead necessary—especially in low-income countries—to encourage their moderate consumption in adherence to recommended daily intakes [15].

## 3. The Environmental Impact of Agri-Food Systems

In the past, the main impact of agriculture on the environment was land occupation, both for cultivation and land pastures, in order to obtain enough food for the growing population; the largest surface area occupied for agriculture was reached in around 1960, approximately 4.8 billion hectares of which 1.5 billion were cultivated (arable land) and the remaining 3.3 billion were pastures and meadows, on a total of 13.3 billion ha available worldwide [17]. Since then the surface area occupied for agriculture has remained almost unchanged, despite a reduction in developed countries and an increase in underdeveloped countries.

In fact:

- in the 2000 years up to 1960 the rise in population from 170 million to 3 billion was followed by an increase in agricultural land to produce the food needed.
- in the last 60 years the “green revolution”, and similar improvements, have increased productivity, enabling the growing population (currently 7.6 billion) to continue to be fed with an almost unchanged area of agricultural land in developed countries, although there are still large areas where low productivity requires the occupation of new land to produce what is needed;
- in some areas of the planet (Amazonia, South-East Asia and Africa) deforestation is still ongoing because wood is needed for work and as domestic fuel, but also because some of these areas are rich in underground resources (oil, gas, minerals etc.);
- finally, a peculiar contribution of agriculture to land occupation is that land is set-aside for bio-fuel production (areas exploited for palm or jatropha oil) and animal feed production (cereals, soybean).

Quite recently Willet et al. [4], stated that: “*agriculture occupies about 40% of global land, and food production is responsible for up to 30% of global greenhouse-gas emissions and 70% of freshwater use*.”, letting the reader suppose that agriculture plays a large part in freshwater shortage and climatic changes, as well as loss of biodiversity and water pollution causing eutrophication and dead zones in lakes and coastal zones.

This way of thinking is now common, especially in people who live far from agriculture; in fact, even Mozaffarian [11] assumed that the food sector is responsible for 25% of the GHG emissions and 80% of the deforestation, that it consumes 32% of the global energy uses and 69% of the freshwater and causes the loss of resilience of our soils and oceans.

However, this is only partly true:

1. agriculture is in fact occupying 37% of the land, globally, but only 12% as arable land;
2. with regard to GHG emission from agriculture, according to IPCC (International Panel Climatic Changes) [18], the total amount of CO2 of agri-food system origin (14.8 Gt/year) represents 25–30% of total anthropogenic CO2, but this can be divided as follows; (i) agricultural emissions 6.2 Gt (10–12%), (ii) land use 4.8 Gt (8–10%), and (iii) beyond the farm gate 3.8 Gt (5–10%). A misunderstanding probably arises from this division, as the whole of the agri-food system does not correspond perfectly to agriculture. In fact, the US Environmental Protection Agency (US EPA) consider agriculture to contribute 9% of the total GHG emissions; ISPRA (Istituto superiore per la protezione e la ricerca ambientale) [19] in Italy have suggested 7%. The contribution to emissions defined as “land use and its change” cannot, indeed, be considered as a normality of agriculture; developed countries’ forestry is currently growing and US agriculture (incl. forestry) is a net sink of C as estimated by the Inventory of U.S. Greenhouse gas emissions and sink, 1990–2016. The quota defined as “beyond farm gate” should also be looked at in more detail: it has a double meaning, indicating flow towards and away from agriculture, the last being perhaps bigger (and not strictly included in agriculture). Therefore, at least in the case of developed countries (which release more GHGs), some 12–14% can be removed from the emissions and the total GHG emissions of true agriculture can be halved. Interestingly, a similar value (14.0%) was shown in the WRI (World Resources Institute) report [17], of which animals accounted for a little over half (about 8.5%);
3. it is true that 70% of the blue water utilized by humans is for agriculture (irrigation), but 60–65% of the world’s food is obtained without irrigation, thanks to green water (rain). Such water is without any other possible use, except plant growth. Therefore, it is meaningless to consider farmed animals as a waste of water, stating that 1 kg of beef needs more than 15,000 L of water. At least for grazing ruminants, the green water (at least 14,414 L, some 93%) is in the soil (from rain) and can only be utilized by plants to produce grazeable (although for humans inedible) plant material or be lost by evaporation.
4. a loss of biodiversity is almost inevitable when new natural surfaces are occupied (particularly for areas that are then cultivated, although much less for those given over to pasture-meadows which represent 2/3 of the occupied land). Nevertheless, biodiversity loss can be lessened by increasing productivity in both cultivated and grazed areas. Phalan et al. [20], in the Spiti Valley in India, have observed that village communities—while improving their livestock yields—have been able to spare land for snow leopards. These results suggest that higher yields are a means to produce more foods and to use them to free up land and conserve biodiversity and ecosystem services.
5. overuse and misuse of nitrogen and phosphorus is certainly a consequence of agriculture intensification, but mainly because of a poorly managed intensive fertilization.

As the above points concern the environmental impact of the entire agri-food system, what can be specifically added for farmed animals? In the world literature there is contradictory information:

- according to Smith et al. [14]: “*… by consuming feedstuffs that people could consume directly, such as grains and legumes, animals reduce the total amount of food available. Today, about half the world’s production of grain is fed to animals, especially monogastrics [21]. Nevertheless, later on the same authors have suggested: “animal-source foods are much more than an expensive and inefficient source of energy. Animals convert low-biological-value protein foods that are less palatable and less nutrient dense to high-biological-value foods that are highly palatable and nutrient dense. This is not conventionally factored into current estimates of the net cost benefit of animal production*”;
- more recently, the FAO [22] report recalled that “*Livestock have received considerable attention in recent years amid controversy about how animal feed production competes for land and other resources with production of human food. Livestock consume a third of all cereals produced and use about 33% of global arable land. They occupy 2 billion ha of grasslands, of which about 700 million could be used to grow crops*.” But later it added that “*the cereals used to feed livestock make up only 13% of their overall diets, with another 1% coming from other human-edible crops. Grass and leaves make up 46% of livestock diets: 19% comes from crop residues; 8% from fodder crops; 5% from oilseed cakes; 5% from other by-products; and 3% from other plant sources that are not edible for humans. Of the plant material fed to livestock, 86% would be inedible by humans directly but is converted into valuable food for human consumption and contributes greatly to food and nutrition security*”.

Apparent contradictions, therefore, arise because although farmed animals are certainly utilizing 25% of the planet’s land for grazing and a small part of arable land for the production of cereals and other foods utilized as feeds, only the latter could be directly consumed by humans, even though a large proportion of animal feed is composed of by-products (brans, oil extraction meals, straws etc.). On the contrary, grazing animals are the only way to exploit the grass of almost natural areas with few human interventions. Therefore, we can say that animal breeding is an opportunity and not a problem; this does not, however, exclude its improvement. In general, there are many ways to progress including, in order of difficulty of implementation: increasing productive efficiency, substitution of inputs with lower impact alternatives and the development of agroecological practices based on the mobilization of biological processes and circularity that often require a redesign of the systems [23]. This last issue could be an extraordinary opportunity, particularly for the improvement of grazed lands, because of both their enormous extension and the scarce attention paid to them in the past. Obviously, this needs caution due to the great differences at the origin and because these areas are essential for biodiversity. In fact:

- Savory [24] showed that grasslands can be enhanced with an intense grazing to sequester enough atmospheric carbon dioxide to reverse climate change. Praised by cattle farmers, his controversial ideas have sparked opposition from other academics. It appears in fact a quite simplistic view, as suggested by Briske et al. [25]: scientific evidence unmistakably demonstrates the inability of Mr. Savory’s grazing method to reverse rangeland degradation or climate change;
- however, in suitable conditions, an improvement is possible [26]: “*When compared with widely used livestock production systems, silvopastoral systems can provide efficient feed conversion, higher biodiversity, enhanced connectivity between habitat patches and better animal welfare, so they can replace existing systems in many parts of the world and should be further developed*.” This system of appropriate intensification can enable a 12-fold reduction of occupied land and a 1.8-fold reduction of methane emissions (per kg of beef);
- finally, as previously suggested by Phalan et al. [20], if some intensification will reduce the total area needed, and a return to natural conditions of those area no longer utilized could improve the natural biodiversity.

A further consideration on the environmental impact of ASFs is that specifically related to less-intensive ASF production on a larger area, using only green (rain) water to grow plants that are then grazed by animals (farmed or wild), because of which only 30% of the blue water utilized for irrigation is attributable to ASF production. Moreover, for GHGs the previously cited WRI report [17] quoted animals as accounting for a little over half of the total agricultural emissions (about 8.5%), and this would not vary significantly if wild animals—also emitting GHG—would return to occupy the currently grazed areas, as has already happened in several European abandoned areas. Capper [27] in fact evaluated the CO2 equivalent emissions of bison herds in 1860 to be double respect to that of all current U.S. dairy cows. However, on the other hand the IPCC [28] calculated that direct livestock GHG emissions, from manure and enteric fermentation, were 2.4 Gt of CO2 equivalent (CO2eq) in 2010, which was about 21% of the total emissions from agriculture, forestry and other land uses, or about 5% of the total anthropogenic GHG emissions (close to the aforementioned 8.5%).

This makes it easier to understand what White and Hall [29] suggested could happen if all farmed animals in the USA were to be eliminated: the modeled system without animals increased total food production (23%), altered foods available for domestic consumption, and decreased agricultural US GHGs (28%), which would represent a 2.6% reduction of the total US GHG emissions. This is of course an improvement, although small, but it does not consider the aforementioned wild animals nor the difficulty in attaining a correct human diet without ASF, because the removal of animals resulted in diets that are non-viable at supporting the nutritional needs of the US population in both the long and the short term without nutrient supplementation.

## 4. Good Animal Health to Improve Productivity and ASF Sustainability

Up to now we have not talked about animal health, but only about the health of man and the planet, yet it is included in the title of this contribution. This is because the magnitude of the linkages and the complexity of the relationships between human health, animal health, nutrition and the environment are extremely important and must be considered within multidisciplinary and interdisciplinary actions of One Health. Such an approach is considered pivotal in designing and promoting policies, strategies and actions for the livestock sector to ensure healthy lives and production efficiency (pp. 26–33, [22]) explaining why the FAO considers its role in ensuring animal health [30] essential, as this in turn contributes to the elimination of hunger, to healthy people and to sustainable food production. The link between human and animal populations, and their relationships with the surrounding environment, is particularly close in developing regions where animals can also provide a mode of transport, draught power, fuel and clothing, as well as proteins (meat, eggs and milk). Animal products also represent a source of income for many small farmers and animal holders in developing countries. On the other hand, the purchase of ASF requires a greater purchasing power, so it’s understandable that economic growth is accompanied by an increase in consumption of animal products and that livestock’s contribution to agricultural GDP is significant in many developing countries [31].

It was already mentioned that human health is guaranteed by mixed diets which include ASF, yet at the same time it is necessary to reduce or restrain the environmental impact of ASF. There are two ways to achieve this: (i) reduce ASF consumption so as to satisfy only the minimum nutritional requirements (but doing so for all human beings) and at the same time (ii) reduce as much as possible the surface area occupied, the GHG emissions and the pollution emitted by farmed animals. As regards the first aspect, we can make some proposals, but the task is for human nutritionists, while for the second aspect proper intensification is of major importance as this will allow an increase in productivity and better efficiency (whether it be for the production meat, milk or eggs).

All this is necessary because, despite agriculture (plant and animal source production) currently having a minor responsibility in the most worrying cause of environmental impact, namely GHG emissions, in the future it will rise. The food needs of the human population are increasing due to the number of people on the planet and the quality of their diet (more ASF); indeed, from 6.7 Gt/y of CO2 in 2010, in 2050 this will reach 9.0 Gt/y if productivity is increased, or 11.2 Gt/y without an increase in productivity [17]. Thus, the WRI [17] report concludes: “*Productivity gains also provide the most important potential synergy between income, food security, and environmental goals.*” Similar statements were made by Balmford et al. [32], suggesting that the additional externalities resulting from the different land requirements of contrasting systems to generate the same quantity of agricultural product are higher for low-yield systems: they require more land (with a reduction of natural habitat) and increase GHG emissions, soil losses etc. Although indirectly, Willet et al. [4] also arrive at the same final considerations: “*However, this Great Food Transformation will only be achieved through widespread, multisector, multilevel action that includes a substantial global shift towards healthy dietary patterns, large reductions in food loss and waste, and major improvements in food production practices.*”

In the case of livestock, in order to increase the efficiency (productivity), in addition to a high genetic merit, optimal feeding, comfortable environmental conditions (dry-soft bedding, protection from excess heat-cold, absence of stressful situations, etc.) and good general management (hygienic-sanitary conditions, reproduction, animal/animal and human/animal interactions, etc.), good health is also essential because altogether they are a guarantee of welfare which, in turn, respond to the ethical responsibility of man toward animals. Ensuring the good health of the animals also reduces human health problems, e.g., zoonosis and food-borne diseases, as well as economic and socio-economic threats like losses in production and profitability, disruptions to local markets and international trade, and last but not least several livelihood threats to the poor (for whom livestock serve multiple functions). However, we must consider that animal health is not only endangered by pathogens and parasites, but also by malnutrition phenomena, which make the individual more susceptible to infection and decreases immune defenses against invading pathogens. In turn, pathogens influence nutritional status, mediated by changes in dietary intake, absorption, and nutrient requirements and losses of endogenous nutrients, all linked to disease.

Malnutrition takes several forms that often appear in combination, such as protein-energy malnutrition and deficiencies in micronutrients such as vitamins A, Fe, Zn, and I. Nevertheless, our interest is also on the effects of malnutrition on tissue damage, metabolic diseases, and digestive disorders, all of which are causes of cytokine release and, consequently, of inflammation and welfare impairment [33].

However, it should be noted that there are big differences in the current spread of diseases (or in the nutritional and management causes of impaired animal health): (1) very little change in the prevalence of endemic diseases in the developing world; (2) a steady decline in the prevalence of many animal diseases in developed countries (despite the increasing prevalence of stress-related diseases, associated with intensive systems). Therefore, although to different extents, and regardless of the cause (infections, parasites or nutrition and metabolic stress), animal diseases all affect human health and life conditions (less ASF available), as well as environment health as lower productivity (for whatever reason) implies more animals, more occupied land, and more GHG emissions and so on.

Since the cause of the impaired health conditions may vary, as may the local socio-economic and technical conditions, so do the ways in which improvements can be achieved:

- in underdeveloped countries technical tools are lacking and only development efforts can improve the situation;
- in developed countries, besides infectious disease prevention, a reduction in production diseases is of major importance, as is the proper use of drugs to avoid risky residues (particularly those causing antimicrobial resistance to antibiotics), but of growing interest is the need to provide acceptable welfare for the farmed animals.

### 4.1. Underdeveloped Countries

A clear example of how important animal health is in developing countries can be found in Ethiopia. With Ethiopia being “…one of the countries with the largest number of livestock in Africa, livestock production plays a major role in the development of Ethiopia’s agriculture. Ethiopian livestock population is estimated to be 59.49 million cattle, 30.697 million sheep, 30.200 million goats, 8 million donkeys, 2.16 million horses, 1.20 million camels, 0.4 million mules and 59.495 million poultry. Nevertheless, development of the livestock sector in Ethiopia is hindered by widespread endemic health problems including bacterial diseases, viral diseases, and parasitic infestation” [34]. Unfortunately, in these countries, there are numerous negative factors, including shortages of veterinary services, lack of technical assistance for animal owners, and the absence of commercial structures both to supply medicines and supplements, but also to enhance animal products (animals, milk, eggs). Other significant negative factors in these countries are a low cultural level and a tendency to have a passive mindset. For this reason, in a recent collaborative paper of mine [35], it was observed that despite several efforts made to introduce advanced technologies into developing countries, very often the results have not been positive. Therefore, I have proposed to involve local people, who must be directly engaged, though also having some external technical and financial support, as currently underway in two pilot centers which are active in Meghalaya (India) and in the province of Lomami (Democratic Republic of Congo). Moreover, the development actions must be compatible with the possibilities of local beneficiaries and must favor a change in their mentality.

Thus the tools to be adopted to facilitate the solution of the problems that make animal breeding difficult in low-income countries are many and varied. Add to this the recommendations of the FAO (pp. 26–33, [22]) to implement animal health programs related to the establishment of best practices in the prevention and control of priority diseases which threaten animal production, public health and trade through its international and regional networks, animal health projects and by disseminating practical information. This because monitoring animal health and preventing animal disease outbreaks is vital to the economy and safety of a country’s food supply, particularly if poor. Thus, healthy animals favor a safer food supply, higher farm productivity (including an increased number of offspring), reduced environmental impact, reduced use of antibiotics and improved animal welfare.

Although promoting animal health and preventing animal diseases is the first step, it must then be followed by everything that enables the production response to be improved: genetics, nutrition, protection from climatic excesses, etc. Unfortunately, however, the aforementioned factors prevalent in low-income countries make this second phase difficult to implement. By nutrition, the extensive systems present in these countries tend to be affected by irregular deficiency problems, which are primarily energy and proteins. These problems occur in hot-dry seasons because of low feed intake, due to the unavailability of grass or the availability of only very low-quality grass. Vitamin deficiencies tend to be a minor problem, yet mineral deficiencies (both macro and trace) can be significant if their soil availability is low, if grass is overly mature and spoiled and if there is any difficulty in providing mineral supplements. Moreover, extensive systems do not exclude problems related to excesses (e.g., minerals in some areas) or the effects of toxicants (e.g., phytoestrogens in subterraneous clover, mycotoxins from Claviceps spp. in fungi-infected grasses, especially fescues, or even toxic weed intake in cases of pasture shortage). Again, all these problems cannot be solved without general development as suggested above.

### 4.2. Developed Countries

At this point, speaking of more or less developed countries, let us remember that infectious-parasitic diseases are of less importance, while production diseases, linked to high production, management errors and stressful factors, take on a greater relevance. They are the cause of reduced welfare and, therefore, of lower productivity. Fortunately, there are many opportunities and existing technologies for increasing the sustainability of the livestock sector through gains in productivity and then efficiency: improvement of genetics, breeding techniques (reproduction), nutrition, general management, manure management and grazing management can contribute to closing yield gaps in all production systems and regions (pp. 95–105, [22]). In a quite recent paper [33], I showed that intensive systems are usually characterized by high feed intake and well-balanced diets. Deficiencies are rare, and excesses are infrequent if the diets are calculated to cover the corresponding requirements of high-yielding animals. This, however, does not exclude the possibility of unsuitable feeding, sometimes of micronutrients but more often of energy and proteins. Of major interest is the case of high-yielding dairy cows that, despite almost doubling their dry matter intake in the first 2–3 months of lactation (by comparison with the dry period), they remain in negative energy balance (NEB) for several weeks, mainly because of the physical limit of the gut size, but also because inflammation can reduce appetite, thus during this period nutrients may be in deficit. Nutrient deficiency and/or unbalance contribute to very early lactation being characterized by an increased risk of metabolic (ketosis) or infectious (e.g., mastitis) diseases.

Nevertheless, with this kind of production system, two further aspects of nutrition–health–welfare relationship are noteworthy:

- defining the best energy (and nutrient) level for the dry period to avoid metabolic diseases and susceptibility to infectious diseases in the transition period;
- managing the diets at the end of pregnancy and at the beginning of lactation to reduce the risks of highly fermentable diets, which help avoid the NEB, but are a possible cause of rumen-intestine fermentation disorders.

The aspect of the ‘fat cow syndrome” in dry period is very important because obese cows at calving time show a very high frequency of retained placenta, milk fever, ketosis, and steatosis, as well as an increased mortality. Furthermore, recent data showed that cows fed a high-energy diet in the dry period, no matter if they are obese or not, appear more susceptible to puerperal metabolic diseases and high liver triglyceride levels, as well as the highest frequency of inflammatory conditions (clinical or sub-clinical). This last observation tends to support the idea that, in ruminants, a relatively prolonged energy excess could trigger a metabolic syndrome like condition (similar to the overweight or obese human situation, characterized by a ‘low level’ inflammation). Therefore, cows fed a high-energy diet in the last part of pregnancy, or which are too fat at calving time, will be at risk of dystocia, retained placenta, ketosis, etc., as well as being more susceptible to infectious diseases [36].

The risk of high fermentability of the diet—that here will be considered for early lactating cows—is, however, typical of any kind of intensive animal husbandry, no matter whether ruminant or monogastric. In both cases, the excesses of starch (and sugars) fermented in the rumen or escaping into the large intestine are responsible for substantial pH reduction (besides an increased and modified microbial population) that tends to increase the endotoxins (lipo-polysaccharides, LPS) or microbe translocation [37]. These phenomena are responsible inter alia for liver abscesses, laminitis and sole ulcers. Nevertheless, the presence of LPS in the rumen and intestine is not always the cause of the appearance of the above health problems, provided that the mucosa is wholesome. An increase of mucosa permeability, due to acidosis itself [38] or to ‘external’ causes (hypoxia, heat stress, heavy physical work, etc. [39]), or to a combination of these factors, can contribute to the above translocation and consequent inflammation with several negative effects.

On the other hand, it is noteworthy the fact that the improvement of sanitary, nutrition and micro-environment conditions ensure better welfare and an improved animal performance in both low-income and developed countries. This makes the role of welfare more important, which is not only justified by ethical reasons, but also by animal productivity. Personal experience with high-yielding dairy cows, often considered as suffering, showed that in intensive dairy farming appropriate management skills are needed, but that high yield can coexist with good welfare conditions for animals [40]. In fact, Buller et al. [41] suggest: “*On the 17 October 2016, the United Nations Committee on World Food Security published its ‘Proposed draft recommendations on sustainable agricultural development for food security and nutrition including the role of livestock*”, recommending animal health and welfare based on the five freedoms and related OIE (World Organization for Animal Health) standards and principles. Health and welfare are closely and mutually related; in the specific case of welfare, it is considered guaranteed when the 5 Brambell freedoms are satisfied. However, their simultaneous insurance seems almost impossible, and it is generally believed that in the intensive agricultural systems of developed countries it is not so easy to respect freedoms 2 and 4 (appropriate environment and normal behavior), while in developing countries it is more difficult to respect freedoms 1 and 3 (from hunger or thirst and pain, injury or disease). Therefore, improving the living conditions (welfare) of animals means satisfying the less respected freedoms as much as possible.

## 5. Conclusions

If we really want to talk about sustainability, we cannot neglect any of the different pillars that comprise it, but in particular the environmental pillar, also with reference to mankind (health of people and society) must operate within correct economic and ethical rules. This means, if a holistic approach is desired, the health of all components must be considered simultaneously: man, animals and planet (One Health). Unfortunately, several recent papers have tended to oversimplify the issue and in particular neglect the true role of agriculture (both plant cultivation and animal breeding), maybe because of a lack of medical and agricultural expertise in their panels. Indeed, some circumstances seem to be overlooked: (i) negative consequences for human health are not only justified by ASF excesses, but also by their deficiencies; (ii) negative effects of agri-food systems on the environment are unavoidable because agriculture tends to compete with nature and foods are irreplaceable; nevertheless, they are often unnecessarily magnified (i.e., by including land-use changes); (iii) the specific contribution of farmed animals from the point of view of GHG emissions and land occupation could be a case of undue magnification because 25% of the planet’s surface must be grazed for several reasons and wild animals could have an impact comparable to that of farmed ones from this point of view.

Nevertheless, it cannot be forgotten that ASF have an ambiguous role: they are supposed causes of non-communicable diseases if in excess, but lack of them is responsible for malnutrition (stunting, anemia, rickets etc. as well as lower cognitive abilities), particularly in low-income countries. For these reasons, we suggest—for ASF consumption—a “motto” from the Roman era: *est modus in rebus*…which means moderation in all things, but that a proper amount of ASF be available for everyone. Furthermore, agriculture and particularly animal breeding has an impact on the environment. Therefore, given that the production of food (plants and animals) must be as low as possible so as to minimize losses and wastes, there remains the fundamental need to use those technologies that reduce this impact. With the same food production (quantity and nutritional quality), a first way to achieve the aforementioned objective is to increase productivity, which can be obtained (according to local conditions) via precision farming, using genetics, management, nutrition and sanitary tools to pursue one inclusive word: health-welfare.

With specific attention to animal health and welfare, it is important to observe that prevention of infectious and parasitic diseases is important but not enough; metabolic diseases and any causes of inflammation must also be prevented in order to improve productivity and welfare. Obviously, the improvement of animal productivity is important in reducing their impact on the environment (fewer animals, lower feed needs, less occupied land, less pollution and lower GHG emissions), but this alone is not enough. Several other aspects in food production, processing and trading are also relevant.

All in all, the hope is that at the base of operational choices there is always science and technology, and that ethics (what is right and why) are never neglected. Humans can in fact use the natural resources, but they have duties towards other human beings and towards nature (environment).
